# CLCF1 Is a Novel Potential Immune-Related Target With Predictive Value for Prognosis and Immunotherapy Response in Glioma

**DOI:** 10.3389/fimmu.2022.810832

**Published:** 2022-02-21

**Authors:** Yuan Jiang, Qiankun Ji, Xiaoyan Long, Peng Wang, Zewei Tu, Xian Zhang, Xingen Zhu, Kai Huang, Jingying Li

**Affiliations:** ^1^ Department of Neurosurgery, The Second Affiliated Hospital of Nanchang University, Nanchang, China; ^2^ Institute of Neuroscience, Nanchang University, Nanchang, China; ^3^ East China Institute of Digital Medical Engineering, Shangrao, China; ^4^ Affiliated Eye Hospital of Nanchang University, Nanchang University, Nanchang, China

**Keywords:** CLCF1, glioma, prognosis, tumor microenvironment, immunotherapy

## Abstract

**Background:**

Cardiotrophin-like cytokine factor 1 (CLCF1) has been described as an oncogene and a potential therapeutic target in a variety of cancers, but its role in glioma remains unknown.

**Methods:**

Based on The Cancer Genome Atlas (TCGA), we conducted a bioinformatics analysis to investigate the clinical significance and biological functions of CLCF1 in glioma at the transcriptional level and predicted the response to immunotherapy of glioma patients with different CLCF1 expression levels. All the results were further verified in Chinese Glioma Genome Altas(CGGA) Data processing and figure generating were performed with R language.

**Results:**

Elevated CLCF1 expression was common in cancers and usually predicted poor prognosis, which was also consistent with gliomas. In Univariate Cox Regression analysis and Kaplan-Meier survival analysis, tumor patients with higher CLCF1 expression tended to experience a worse prognosis. In the multivariate Cox proportional hazard model, the expression of CLCF1 was an independent prognostic factor in gliomas. The biological function analysis of CLCF1 in glioma showed that CLCF1 was closely associated with immune signatures, including immune-related pathways, immune cell infiltration, and immune checkpoints. Moreover, glioma patients with low CLCF1 expression showed a greater tendency to respond to anti-PD1/PD-L1 immunotherapy, indicating CLCF1 also had potential clinical significance in guiding immunotherapy. And CLCF1 as a member of the IL6 family had a better predictive value for prognosis and immunotherapy response in glioma than that of IL6 and other IL6 family members.

**Conclusion:**

CLCF1 expression is an independent prognosticator and a promising therapeutic target correlated with immunotherapy in glioma.

## Introduction

Gliomas are the most common and lethal malignant central nervous tumors, the therapeutic effects of currently available conventional treatment including surgical resection, chemotherapy, and radiotherapy are limited (1, 2). Given the pivotal role of immunity in the initiation and progression of tumors demonstrated in recent studies, immunotherapy represented by PD-1/PD-L1 inhibitors (3) has provided novel choices for the treatment of glioma. However, due to the complex inhibitory immune microenvironment and systemic immunosuppression of glioma, the curative efficacy of the currently available immunotherapy in gliomas is unsatisfactory (4, 5). Therefore, the search for new and effective biomarkers and therapeutic targets related to immunity is still helpful in promoting glioma treatment. IL-6 cytokine (glycoprotein 130) family is known for its high degree of functional pleiotropy and redundancy (6). All cytokine family members are characterized by the usage of common β-receptor signaling subunits, and transmit intracellular signals through the JAK-STAT, MAPK, and PI3/Akt signaling pathways (7, 8). In the healthy organism, the IL-6 cytokine family plays a notable role in regulating immune homeostasis, inflammation, development, and metabolism. In cancers, the IL-6 cytokine family not only directly affects the proliferation, survival, invasion, and metastasis of tumor cells, but also participates in shaping the local tumor microenvironment (TME) by modulating inflammation, immunosuppression, and angiogenesis (9, 10). Moreover, The autocrine and paracrine characteristics of the IL6 family may be related to the systemic immunosuppression of glioma (11–14). Thus, the dysregulation of IL-6 family cytokine expression and the corresponding receptor signaling pathways is a common phenomenon in cancers and is often associated with adverse clinical outcomes (15). As a member of the IL-6 cytokine family, CLCF1 is known as a potent neurotrophic factor, B-cell stimulatory agent, and a neuroendocrine modulator of pituitary corticotropic cell function (16). The deficiency of CLCF1 function causes cold-induced sweating syndrome (CISS), a disease associated with respiratory and neural developmental defects (17). Currently, the known role of CLCF1 in tumors is mainly related to the immune microenvironment. In hepatocellular carcinoma, CLCF1 levels in cancer-associated fibroblasts (CAFs) promote tumor cell stemness as well as the infiltration and polarization of tumor-associated neutrophils (TANs) (18). In lung cancer, CLCF1 is also a critical protumorigenic factor for CAFs (19), and an engineered decoy receptor targeting CLCF1-CNTFR signaling was confirmed to induce anti-tumor activity in lung adenocarcinoma (20). As an IL-6 family cytokine with the neurodevelopmental function that participates in the regulation of the tumor immune microenvironment, CLCF1 has also been found to be a promising prognostic marker and target associated with PTEN mutation in glioma in a recent study (21), but its specific role remains to be further explored. In the present study, we identified the prognostic significance of CLCF1 in gliomas based on RNA-sequencing data and clinical data of glioma patients extracted from The Cancer Genome Atlas (TCGA) and the Chinese Glioma Genome Atlas (CGGA). Next, we conducted Gene set variation analysis (GSVA) to investigate the associated biological processes and pathways. In addition, we performed a comparative analysis of the differences in the proportion of immune infiltrating cells, immune checkpoints expression, and predicted response to immunotherapy between the subgroups. The predicted immunotherapeutic response indicated that patients with low CLCF1 expression would achieve a significant benefit from anti-PD-1/anti-PD-L1 immunotherapies.

## Materials And Methods

### Data Acquisition

In the study, clinical information and gene expression data used in the pan-cancer analysis were acquired from publicly available databases. The CLCF1 expression data and corresponding clinical data in 33 cancers were extracted from TCGA and partial data from normal tissue derived from Genotype-Tissue Expression (GTEx).

The two independent glioma cohorts used in the study, including TCGA and the CGGA cohorts. The relative expression data and clinicopathological information of the TCGA cohort were acquired from the Genomic Data Commons Data Portal website (GDC; https://portal.gdc.cancer.gov/), while data relative to the CGGA cohort in the present study were retrieved from the CGGA website (http://www.cgga.org.cn/).

### Patient Exclusion Criteria

The criteria for including patients in the two independent glioma cohorts were as follows: (a) primary glioma patients with overall survival (OS) > 1 month; (b) patients with mRNA sequencing data; and (c) complete WHO Grade classification data for each patient. With these inclusion criteria, we obtained 607 samples from the TCGA database and 961 samples from the CGGA database. The summary of basic information for the selected patients is presented in **Supplementary Table S1**. However, in the pan-cancer analysis of CLCF1, to maintain the consistency among various tumor standards, primary glioma patients with overall survival (OS) ≤ 1 month were also included in the analysis.

### Data Processing

The Transcripts Per Kilobase Million (TPM) values were transformed from The Fragments Per Kilobase of transcript per Million (FPKM) data values of the two glioma cohorts with the same algorithm described in previous studies (22, 23), which were used in subsequent experimental analysis. The CGGA cohort data consisted of CGGA629 and CGGA325 datasets. The batch effect between TCGA, CGGA629, and CGGA325 datasets was eliminated with the R package “SVA” (24).

### Functional Annotation and Gene Set Variation Analysis

Samples were divided into high and low expression subgroups based on the median CLCF1 expression level, then GSVA was performed (25) to single out the enriched pathways based on Gene Ontology (GO) terms (c5.go.bp. v7.4. symbols) and Kyoto Encyclopedia of Genes and Genomes (KEGG) analysis (c2.cp.KEGG.v6.2. symbols) genesets with the criteria P<0.05, |log2 (foldchange)| >0.2). Gene set enrichment analysis (GSEA) software (version 4.0.1, https://www.gsea-msigdb.org/gsea/index.jsp) (26) was also used to perform GSEA analysis to identify Hallmark pathways enriched in the high CLCF1 expression subgroup. The tumor hallmark pathways were filtered out using the |Normalized Enrichment Score (NES)| > 1.5, normalized P-value <0.05, and false-discovery rate (FDR) q-value <0.25.

### Immunological Features Related Analysis in Glioma

We selected 29 representative “immune signatures”, which represented diverse immune cell types, functions, and pathways, and calculated the enrichment score of each sample using single-sample GSEA (ssGSEA) according to the method described by Xu et al (27), then arranged the order of each sample according to the expression of CLCF1, and plotted the heatmap according to the corresponding ssGSEA score. To further analyze the TME of glioma, The ESTIMATE algorithm was utilized to evaluate the abundance of immune cells (ImmueScore) and stromal cells (StromalScore), as well as the abundance of non-tumor composites (ESTIMATEScore) (28). The infiltration level of 22 types of tumor-infiltrating immune cells (TIICs) in the TME of each sample was calculated *via* a deconvolution method according to linear support vector regression (CIBERSORT) (29), and the statistical differences of TIICs in subgroups were obtained by comparison. We also collected 27 immune checkpoints (ICPs) with therapeutic potential based on the study by Auslander et al (30, 31), and verified their correlation with CLCF1 expression.

### Construction and Validation of the Nomogram Model

The nomogram model was constructed using the “rms” R package, by fitting common significant prognostic factors (CLCF1 expression and Grade) derived from TCGA and the CGGA datasets to predict the OS of glioma patients. To verify the accuracy and reliability of the model, the calibration plots and decision curve analysis (DCA) were constructed.

### Prediction and Verification of Immune-Checkpoint Blocker (ICB) Therapy Response

To predict the clinical response of each patient to immunotherapy, the expression profiles of glioma patients were analyzed using the TIDE website tool (Tumor Immune Dysfunction and Exclusion; http://tide.dfci.harvard.edu/) to determine the TIDE score (32). The predicted immunotherapeutic response associated with CLCF1 was further validated in the IMvigor210 cohort (anti-PD-L1-immunotherapy for urothelial cancer) (33) and the GSE78220 cohort (anti-PD-1-immunotherapy for metastatic melanoma) (34). The data of the two cohorts were downloaded from IMvigor210 (http://research-pub.Gene.com/IMvigor210CoreBiologies) and the GSE78220 dataset in GEO.

### Western Blotting and Antibodies

We collected 3 normal brain tissue samples and 6 glioma samples (3 LGG, 3 GBM) for this study, all tissue samples came from the Second Affiliated Hospital of Nanchang University and were collected from December 2020 to June 2021.

After homogenization, the tissue samples were lysed with RIPA cell lysis buffer, and the protein lysate was obtained after high-speed centrifugation. For western blotting (WB), lysates were separated by 12% SDS-PAGE and transferred to PVDF membranes to incubate with primary antibodies, including Vinculin (1:2000, Proteintech) and CLCF1 (1:5000, GeneTex), followed by further incubation with the corresponding secondary antibodies, the bands on the membranes were finally visualized using the enhanced chemiluminescence (ECL) substrate (Thermo) using the GV6000M (GelView 6000pro) Automatic Chemiluminescence Imaging System. The intensity of the protein bands was quantified by ImageJ software and standardized according to the levels of beta-Tubulin.

### Quantitative Real-Time PCR

The tissue samples used here were the same as those obtained for the WB studies described above. Total RNA was extracted from brain tissue and reverse-transcribed into complementary DNA. Next, relative mRNA expression of genes was normalized to that of beta-tubulin, and the fold change was evaluated using the 2−ΔΔCT method. The primer sequences used for quantitative Real-Time PCR (qRT-PCR) were obtained from RiboBio (Guangzhou, China) and were as follows: CLCF1 forward 5′-CTTAGCTGGGACCTACCTGAA-3′, reverse 5′-CCACACTTCCAAGTTGACCGT-3′; and Tubulin beta forward 5′-GGCCAAGGGTCACTACACG-3′, reverse 5′-GCAGTCGCAGTTTTCACACTC-3′.

### Immunohistochemistry

The immunohistochemistry of pathologic specimens of the CLCF1 was downloaded from The Human Protein Atlas (www.protein.atlas.org/) and contained the quantity and intensity of staining and the relative clinical data of patients.

### Statistical Analysis

The two-sided log-rank test was used to compare the clinical prognosis between the low- and high- CLCF1 expression subgroups with Kaplan-Meier (KM) curve analysis, and the reliability of prediction was tested with receiver operating characteristic (ROC) curves. The prognostic role of CLCF1 expression was evaluated by univariate and multivariate Cox regression analysis. Correlations between variables were assessed by Spearman’s or Pearson’s correlation analyses. Variables with a normal distribution were analyzed by the unpaired Student’s t-test. All statistical analyses were performed *via* the R program language (version 3.6.1, https://www.r-project.org/) in this study.

## Results

### Pan-Cancer Analysis of CLCF1 and High CLCF1 Expression Predicted Poor Prognosis in Gliomas

**Figure 1:**
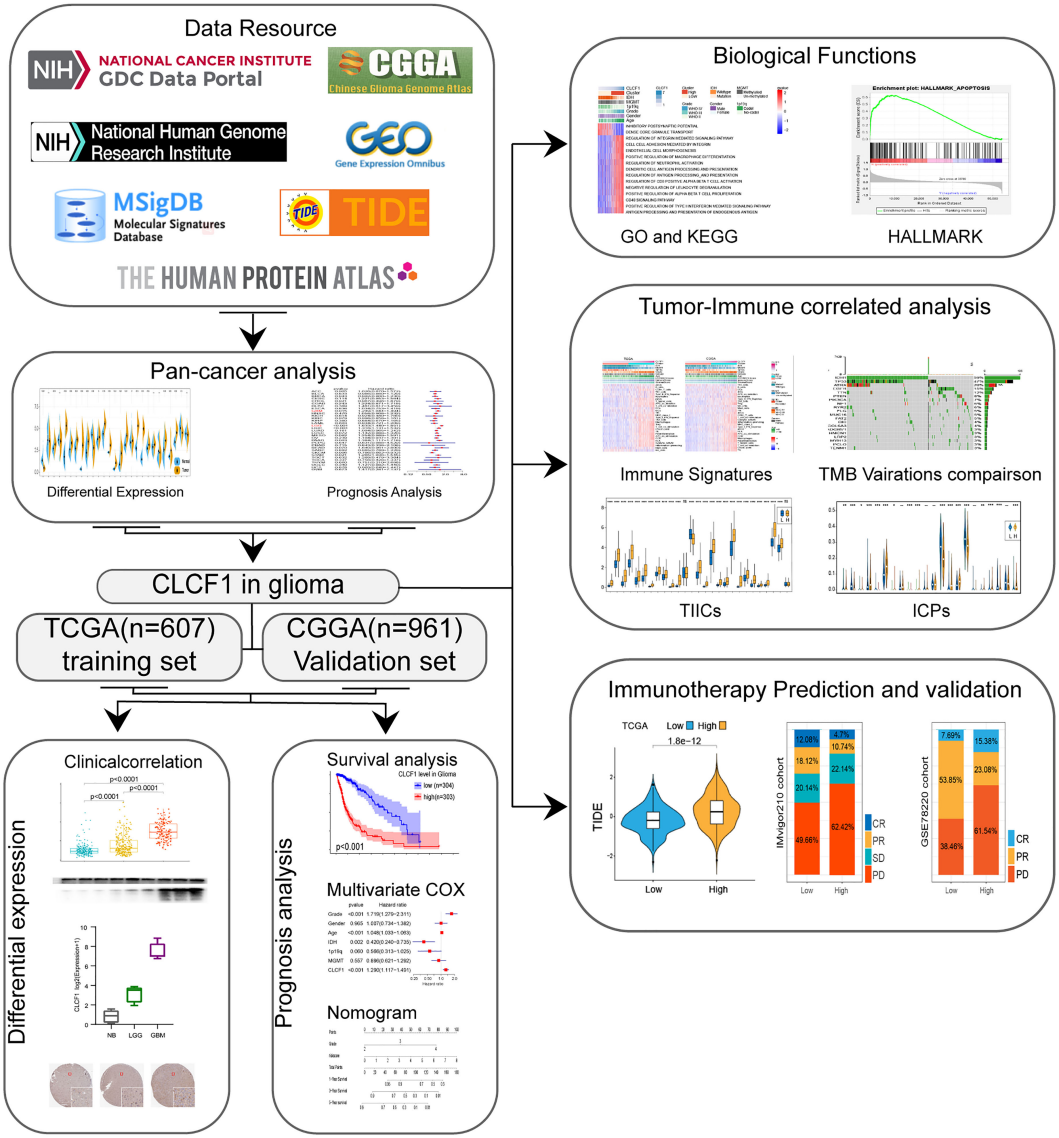
Study flow chart.

**Figure 2:**
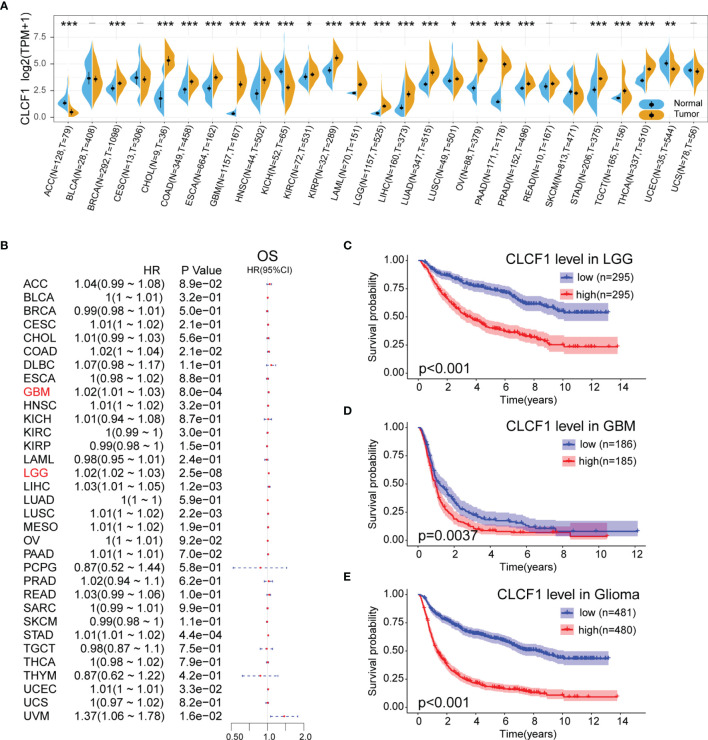
**(A)** Comparison of CLCF1 expression in different tumor tissues and corresponding normal tissues, Significance: - P > 0.05, *P < 0.05, **P < 0.01, ***P < 0.001; **(B)** Univariate Cox Regression analysis of CLCF1 expression and clinicopathological characteristics in different cancers; Kaplan-Meier analysis of CLCF1 in **(C)** LGG, **(D)** GBM, and **(E)** Pan-glioma based on the TCGA dataset.

The flow chart of our study process is shown in **Figure 1**. Aberrant expression of CLCF1 in human cancers was found by comparing the pan-cancer data extracted from TCGA and GETx databases (**Figure 2A**). The results of the comparison indicated that the CLCF1 expression was obviously higher in 17 tumors, including BLCA, BRCA, CHOL, COAD, ESCA, GBM, HNSC, KIRP, LAML, LGG, LIHC, LUAD, OV, PAAD, PRAD, STAD, TGCT, THCA; and slightly higher in KIRC and LUSC. However, CLCF1 expression was lower in ACC, KICH, UCEC.

To analyze the prognostic value of CLCF1 expression in the 33 cancer types, We conducted univariate Cox Regression analysis to investigate the correlation between CLCF1 expression and OS (**Figure 2B**), disease-free survival (DFI) (**Supplementary Figure S1A**) and disease-specific survival (DSS) (**Supplementary Figure S1B**), in TCGA. The detailed results are displayed in Forest charts. On visual inspection, high expression of CLCF1 was inversely correlated with OS in COAD (p=0.021), GBM (p<0.001), LGG (p<0.001), LIHC (p=0.0012), LUSC (p=0.0022), STAD (p<0.001, UCEC (p=0.033); inversely correlated with DSS in COAD (p=0.0063), GBM (p=0.0013), LGG (p<0.001), LUSC (p<0.001), STAD (p=0.04), UCEC (p=0.0045), UVM (p=0.028); and inversely correlated with DFI in LGG (P=0.022), LUSC (p=0.019), PAAD (p=0.016), PRAD (p=0.0093), UCEC (p=0.0084).

According to the above results, high expression of CLCF1 in both LGG and GBM indicated a similar negative effect on prognosis. To further assess the prognostic value of CLCF1 in glioma, we grouped patients into high and low CLCF1 expression subgroups to conduct KM survival analysis. The results indicated that patients with higher CLCF1 expression achieved worse OS prognosis in LGG and Pan-glioma in the TCGA cohort (**Supplementary Figures S1C–E**), and in the CGGA cohort, high CLCF1 subgroup achieved worse OS prognosis in LGG, GBM, and Pan-glioma (**Figures 2C –E**). The Receiver operating characteristic (ROC) curves were also constructed to evaluate the predictive efficiency of KM curves (**Supplementary Figures S1F–K**).

### Correlation Between CLCF1 and Clinicopathological Characteristicsin Pan-Glioma

**Figure 3:**
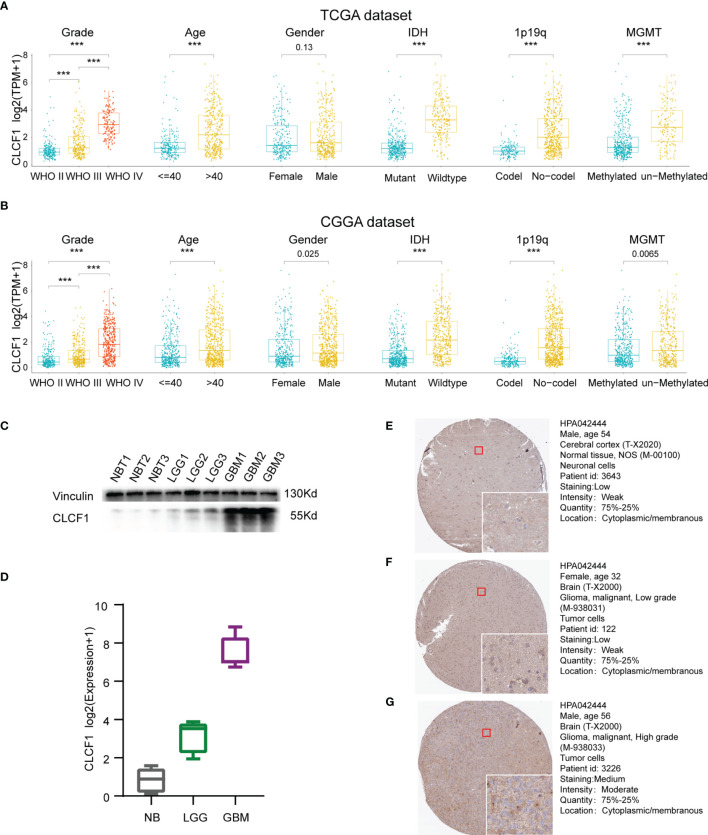
Correlation between CLCF1 and WHO grade, age, sex, IDH mutation status, 1p/19q co-deletion status, and MGMT promoter methylation in **(A)** TCGA and **(B)** CGGA datasets, Significance: ***P < 0.001; **(C)** Western blotting analysis; **(D)** qRT-PCR analysis; IHC staining for CLCF1 in **(E)** normal brain tissue, **(F)** LGG tissue, and **(G)** GBM tissue.

Isocitrate dehydrogenase (IDH) mutation, 1p/19q codeletion status, and O6-methylguanine DNA methyltransferase (MGMT) promoter methylation have all been well demonstrated to relate with the malignancy of gliomas. Given its important role in glioma, these molecular biomarkers were also included in the clinicopathological characteristics. As shown in the boxplots, in the TCGA dataset (**Figure 3A**), CLCF1 expression was statistically significantly elevated along with the WHO grade level. Patients with older age, IDH wildtype status, 1p/19q no-codel status, or MGMT un-methylated status were also associated with higher CLCF1 expression. All these correlations also existed in the CGGA dataset (**Figure 3B**).

### Validation of Transcription and Protein Expression Levels of CLCF1 in Gliomas

To further confirm the differences in expression of CLCF1 in glioma and normal brain tissues, we used WB and qRT-PCR to detect protein (**Figure 3C**) and gene expression transcription levels (**Figure 3D**) in the samples. We found that the mRNA and protein expression of CLCF1 were the lowest in normal tissues and increased with grade in glioma samples. As for the immunohistochemistry of pathologic specimens of the CLCF1 (**Figures 3E** **–G**), the quantity and intensity of staining were also consistent with the above results.

### Independent Prognostic Value of CLCF1

**Figure 4:**
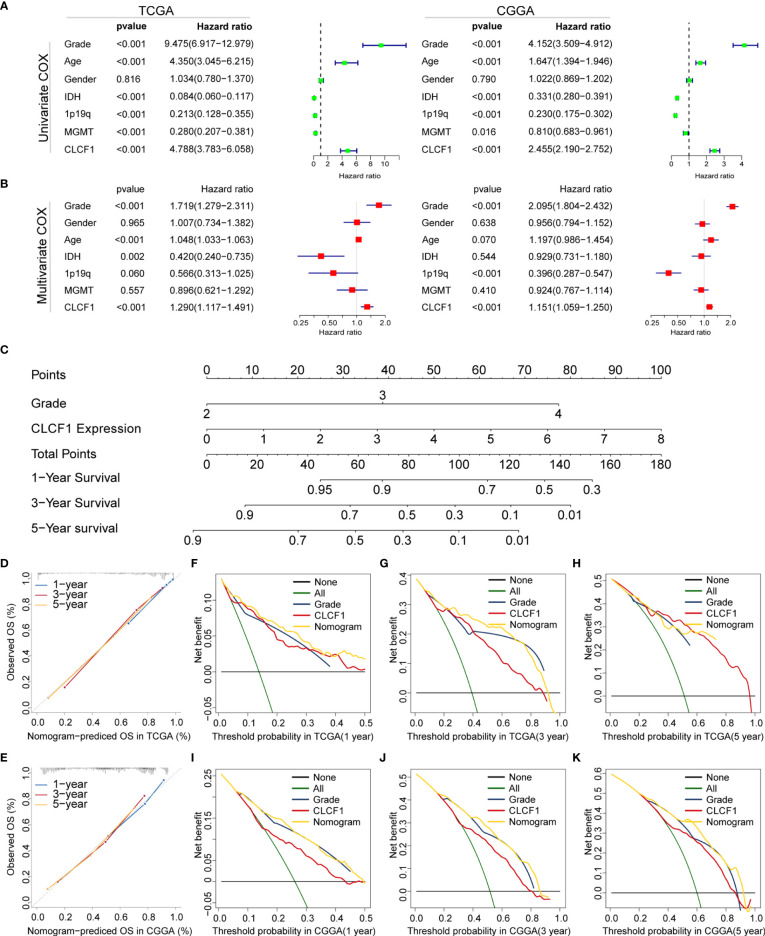
Univariate and multivariate Cox analysis of clinicopathological characteristics and CLCF1 expression in **(A)** TCGA cohort and **(B)** CGGA cohorts; Nomogram constructed with WHO grade and CLCF1 expression in **(C)** TCGA cohort; **(D, E)** The calibration plots: verify the accuracy of the predicted 1-, 3- and 5- OS in the nomogram; and **(F–K)** DCA curves: CLCF1 and Grade curves represent its own prognostic value, Nomogram curve represents the synthetical prognostic value of CLCF1 and Grade, None curve represents no prognostic value and All curve represents the theoretical best prognostic value.

In combination with the above results, we found CLCF1 showed strong correlations with clinicopathological characteristics in pan-glioma. To investigate whether CLCF1 may be considered an independent prognostic factor in gliomas, univariate and multivariate Cox regression analysis was performed. On univariate Cox analysis (**Figure 4A**), the WHO grade, age, IDH mutation status, 1p/19q deletion status, MGMT promoter methylation status, and CLCF1 expression were closely related to the prognosis of glioma in TCGA and CGGA datasets. On multivariate COX analysis (**Figure 4B**), the WHO grade, age, IDH status, and CLCF1 expression were independent prognostic factors in the TCGA dataset; while in the CGGA dataset, tumor grade, age, 1p/19q status, and CLCF1 expression were independent prognostic factors. This difference may be related to the fact that samples with incomplete grade information had been completely removed, while samples with incomplete information such as 1p19q, IDH mutations, and MGMT still retained in the datasets. Of note, the independent prognostic value of CLCF1 could also be applied separately in LGG and GBM (**Supplementary Figure S2**).

### Construction and Validation of Nomogram

To further evaluate the prognostic prediction ability of CLCF1 and its potential in clinical application, we extracted grade and CLCF1 expression, having an independent prognostic value in both TCGA and CGGA datasets, to develop a nomogram model for OS prediction. Grade and CLCF1 expression were assigned corresponding scores and the total scores could be used to predict OS (**Figure 4C**).

In the calibration curves for the TCGA training cohort (**Figure 4D**), the predictive curve achieved high accuracy in forecasting the 1-, 3-, and 5-year OS of glioma patients (C-index 0.831). As for the CGGA verification cohort (**Figure 4E**), the calibration curves maintained considerable accuracy (C-index 0.741). To compare the clinical usefulness of grade and CLCF1 expression based on the threshold probability and verify the reliability of this nomogram model, we constructed the DCA curves. In the TCGA training cohort (**Figures 4F** **–H**), CLCF1 expression displayed excellent applicability and retained relative validity in the CGGA validation cohort (**Figures 4J** **–K**). With regard to the nomogram, it was also an excellent predictive evaluation model and was superior to tumor grade or CLCF1 expression level alone in predicting OS.

### Biological Function of CLCF1 in Glioma

**Figure 5:**
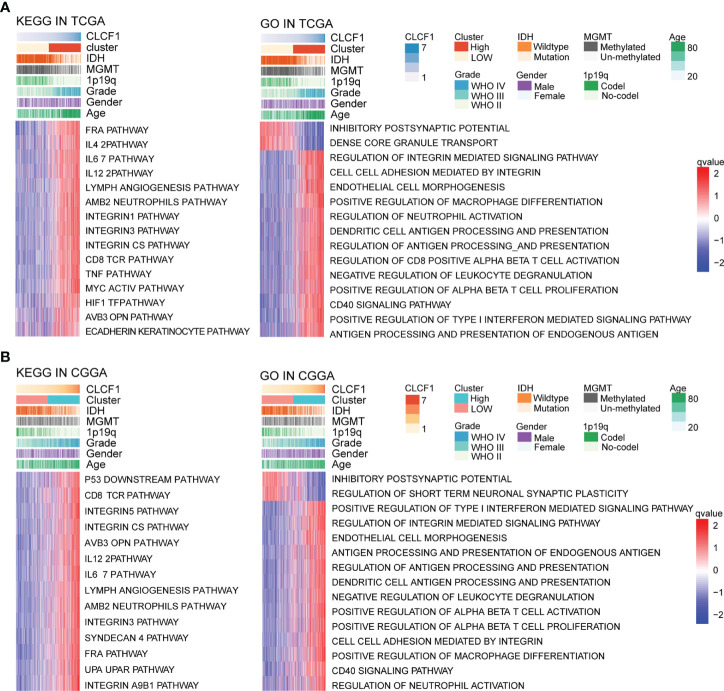
KEGG and Gene Ontology analysis for CLCF1 in pan-glioma based on **(A)** TCGA and **(B)** CGGA datasets.

To explore the potential role of CLCF1 in the occurrence and development of pan-glioma, we performed GSVA using the TCGA (**Figure 5A**) and CGGA (**Figure 5B**) datasets. Detailed results are listed in **Supplementary Tables S2**, **S3**. In both TCGA and CGGA datasets, high CLCF1 expression was associated with hyperactivated pathways correlated with immunity, tumorigenesis, and the extracellular matrix, including antigen processing and presentation, cell adhesion, and regulation of oncogenes. In particular, the integrin-related multiple oncogenic pathways showed a robust correlation with CLCF1. Similar enrichment analysis results were also seen in independent analyses of LGG (**Supplementary Figure S3**) and GBM (**Supplementary Figure S4**), suggesting that CLCF1 may be involved in all grades of glioma and played a similar role in promoting cancer. In addition, the activities of some normal biological processes were contrary to the trend of CLCF1 expression including cell differentiation, and normal cell division activities were inhibited. We also conducted GSEA analysis and filtered out 35 cancer hallmark gene sets significantly enriched in pan-glioma with having a ClCF1 high expression phenotype with the criterion of a normalized enrichment score >1.5, and identified apoptosis, coagulation, glycolysis, hypoxia, epithelial_mesenchymal_transition, and p53_pathway gene sets (**Supplementary Figures S5A–H**). Detail results are listed in **Supplementary Table S4**.

### The Relationship Between CLCF1 and Immunity in Gliomas

**Figure 6:**
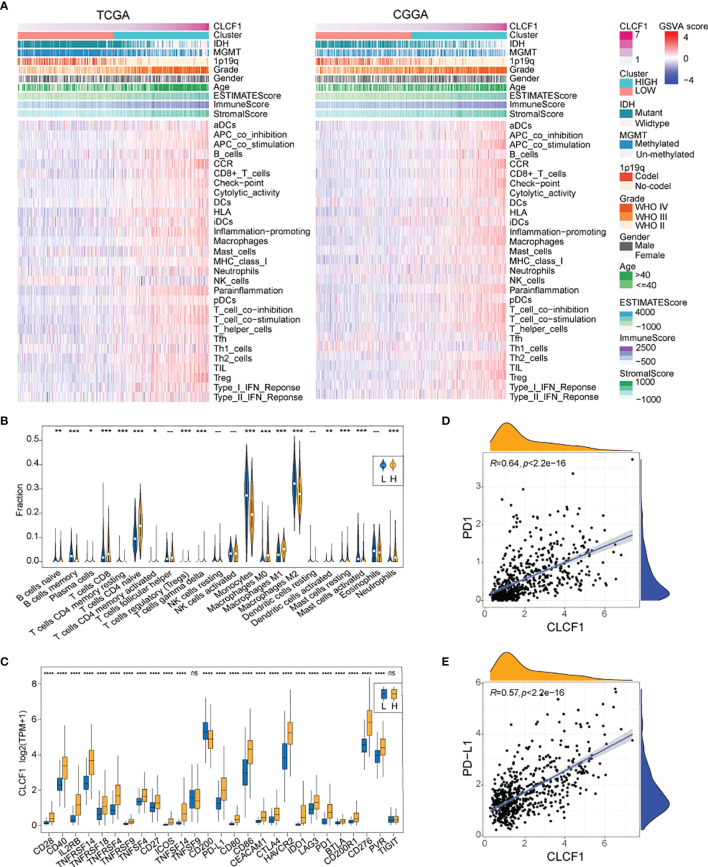
**(A)** The heatmap for enrichment scores based on “immune signatures” in TCGA and CGGA; **(B)** Comparison of TIICs between the low and high CLCF1 expression groups in TCGA; **(C)** The difference of ICPs expression between subgroups in TCGA; The correlation between expression of CLCF1 and **(D)** PD1, **(E)** PD-L1 and in TCGA cohort; Significance: ns P > 0.05, *P < 0.05, **P < 0.01, ***P < 0.001, ****P < 0.0001.

The biological function analysis described above revealed that CLCF1 may play an important role in tumor immunity. In the heatmap of “immune signatures” (**Figure 6A**), CLCF1 expression presented a strong positive correlation with most immune signatures, including the para-inflammation, inflammation-promoting, macrophages, T cell co-stimulation, HLA, check-point, and CCR (**Supplementary Figures S6D–J, N–T**). Meanwhile, the high CLCF1 expression subgroup achieved a significantly higher immuneScore, stromalScore, and Estimatescore (**Supplementary Figures S6A–C, K–M**), which implied samples in the high CLCF1 expression subgroup contained greater immune cell infiltration and stromal cells. Similar results can also be seen in the separate analysis of LGG and GBM (**Supplementary Figure S7**).

As for the relationship between CLCF1 expression and tumor-infiltrating immune cells (TIICs), We compared the analytical results of TCGA (**Figure 6B**) and CGGA (**Supplementary Figure S8A**) and found a similar statistically significant difference in the distribution of plasma cells, CD8+ T cells, naïve CD4+ T cells, activated memory CD4+ T cells, T regulatory cells (Tregs)(**Supplementary Figure S8E**), gamma-delta T cells, monocytes, M0 macrophages, and M1 macrophages. As for the 27 immune checkpoints, we compared the expression differences of ICPs in CLCF1 high and low subgroups in TCGA and CGGA respectively (**Figure 6C** and **Supplementary Figure S8B**). Most ICPs have higher expression in the high CLCF1 subgroup, such as CD279 (PD-1) and CD274 (PDL-1), which were positively correlated with CLCF1 expression (**Figures 6D, E** and **Supplementary Figures S8C, D**). Similar results also exist when the comparison was executed in the separated LGG and GBM datasets (**Supplementary Figure S9**), which indicated that CLCF1 may played a stable role in the immune process of glioma.

### Correlation of CLCF1 Expression With Prediction of Response to Immunotherapy

The efficacy of anti-PD1 immunotherapy in glioma patients can be influenced by the tumor mutation burden (TMB), PD-L1 (CD274) expression, and tumor immune microenvironment cell components(TICC) (35–38). Altogether our findings indicated that CLCF1 was positively correlated with Treg levels (**Figure 6B** and **Supplementary Figure S8E**) and check-points immune signatures (**Figure 6A**), which drove us to investigate the association between CLCF1 expression and immune response. In the comparison between the two subgroups, the expression of most immune checkpoints proteins was significantly increased in the high CLCF1 expression subgroup, including PD1 (**Figure 6D**) and PD-L1 (**Figure 6E**).

**Figure 7:**
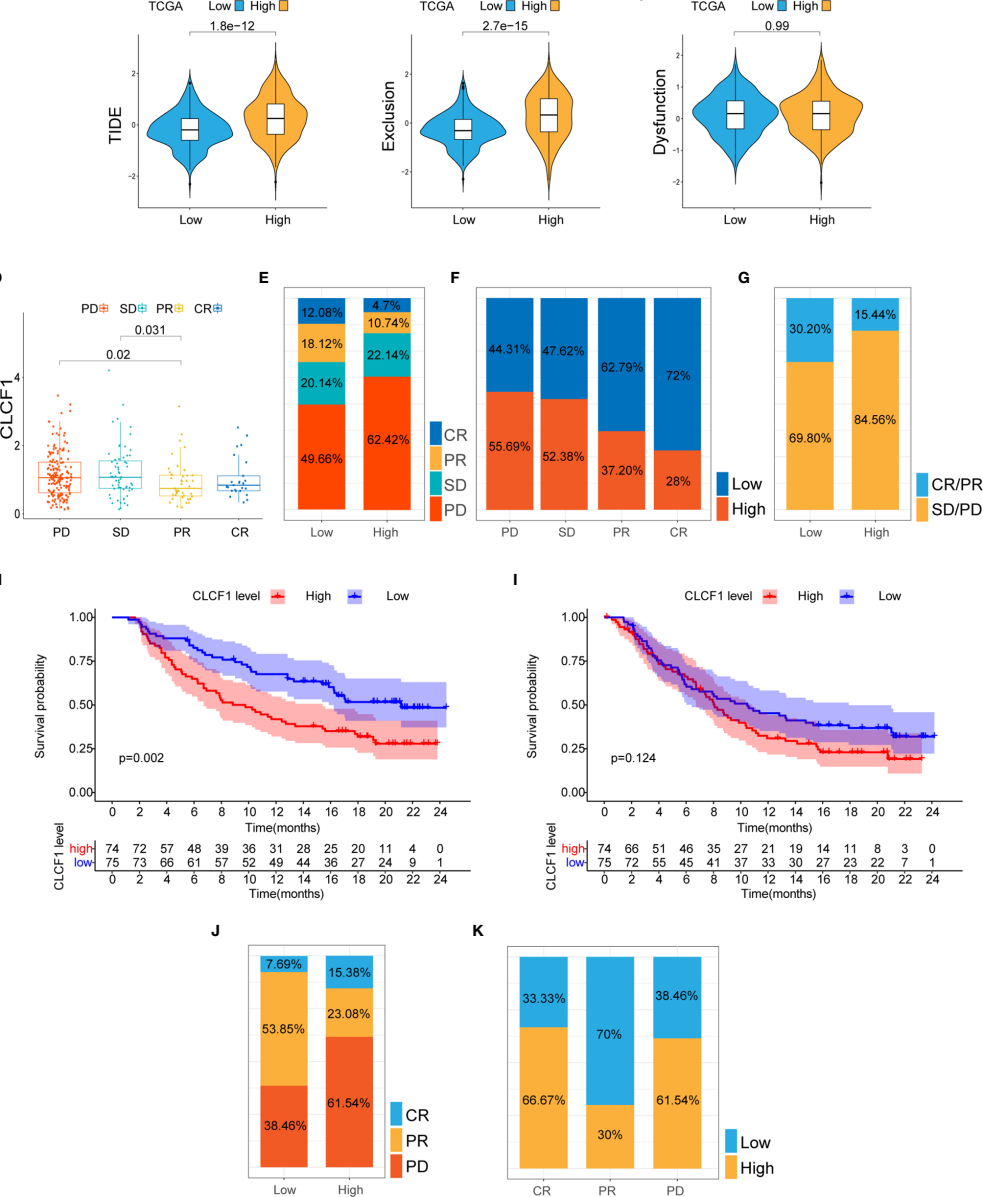
**(A–C)** The TIDE, T cell exclusion, and dysfunction score in low and high CLCF1 subgroups in TCGA; **(D)** Expression difference of CLCF1 in different anti-PD-L1 clinical response groups in the IMvigor210 cohort; The proportions of various immunotherapy response types in the high and low CLCF1 expression subgroups in the IMvigor210 cohort **(E)** and the GSE 78220 cohort **(J)**; The proportion of high and low CLCF1 expression correlated with clinical immunotherapeutic response subgroups in the IMvigor210 **(F)** and GSE 78220 **(K)** cohorts; **(G)** The proportions of clinical responses to anti-PD-L1 immunotherapy in the high and low CLCF1 groups in the IMvigor210 cohort; Kaplan-Meier survival plot of CLCF1 in the high PD-L1 group **(H)** and low PD-L1 group **(I)** in the IMvigor210 cohort.

As for TMB in pan-glioma, the high CLCF1 expression subgroup presented higher somatic mutation levels (**Supplementary Figure S10C**) and higher mutation levels in TP53, ATRX, EGFR, and PTEN, while the low CLCF1 subgroup presented higher mutation level in IDH1 and CIC(**Supplementary Figures S10A, B**). Based on the above results, we used the TIDE algorithm to predict the response of glioma patients to ICBs (anti-CTLA-4 and anti-PD-1) therapy. The results indicated that patients in the high CLCF1 expression subgroup achieved higher scores in TIDE (**Figure 7A**) and Exclusion (**Figure 7B**), which indicated that the patients could achieve less sensitivity to ICB treatment because of T cell exclusion (32). While the scores in Dysfunction (**Figure 7C**) got no statistical significance between the subgroups. The above results were consistent in both TCGA and CGGA cohorts (**Supplementary Figures S11A–C**). The specific TIDE scores are listed in **Supplemenaryt Tables S5**, **S6**. Verification of the prediction of response to ICBs treatment in both the IMvigor210 and GSE78220 cohorts also supported the above conclusion. In the IMvigor210 cohort, the expression levels of CLCF1 and PD-L1 clearly had an impact on the overall survival of patients(**Supplementary Figures S11D, E**). CLCF1 expression in partial response group was significantly lower than that in progressive disease (PD) and stable disease (SD) patient groups (**Figure 7D**), and patients predicted to be sensitive to ICBs treatment accounted for a larger proportion in the low CLCF1 expression subgroup (**Figures 7E –G**), which was also verified in the GSE78220 cohort (**Figures 7J –K**). We further divided the IMvigor210 cohort samples into high-PD-L1 and low-PD-L1 groups according to the expression of PD-L1 and conducted KM survival analysis stratifying by CLCF1 expression, respectively. The survival difference in the high-PD-L1 expression group (**Figure 7H**) was more marked than that in the low PD-L1 group (**Figure 7I**), which suggested that CLCF1 expression exerted a synergistic effect with anti-PD-L1 treatment.

### Comparison of Clinical Significance Among IL-6 Family Members

Next, we collated the p-values from the univariate Cox regression analysis and Kaplan-Meier analysis associated with the expression of the IL-6 family members in gliomas (**Supplementary Tables S7**, **S8**). The results indicated that CLCF1 achieved broader and more reliable prognostic ability than IL-6 in gliomas. In addition, the proportion of anti-PD-L1 immunotherapy responses in the low- and high-expression subgroups of the IL-6 family members in the imvigor210 cohort (**Supplementary Table S9**) indicated that CLCF1 expression had the highest sensitivity to anti-PD-L1 immunotherapy.

## Discussion

We comprehensively analyzed the correlation between CLCF1 expression, prognosis, clinicopathological features, tumor mutations, and tumor immunity based on publicly available datasets of clinically characterized glioma patients. The results suggested that the expression of CLCF1 was an independent prognostic factor among the relevant clinical features of glioma and possessed the potential to be a target associated with response to immunotherapy.

To accurately evaluate the prognostic significance of CLCF1 in glioma, We performed survival analysis in LGG, GBM, and pan-glioma respectively. In the subsequent functional analysis, we scored all glioma samples based on GO biological processes, KEGG pathways, and immune signatures *via* the GSVA algorithm using an unsupervised and nonparametric approach. The composition of TME and TIICs of all samples were also evaluated by ESTIMATE and CIBERSORT algorithms. All samples were divided into high and low subgroups according to the expression of CLCF1, and the results were obtained from a comparison between the subgroups. To increase the reliability of the results, all analyses were performed in both TCGA and the CGGA cohorts, respectively, and the results were confirmed in both datasets. Our findings indicating response to immunotherapy for the different subgroups predicted by the TIDE algorithm were further validated using the IMvigor210 cohort and the GSE78220 cohort.

In recent years, immunotherapy represented by anti-PD-1 therapy has shown promising therapeutic potential in partial preclinical research of glioma (39, 40), but most immunotherapy clinical trials have failed to achieve the expected treatment efficacy (41). The complex immunosuppressive TME of glioma may be one of the primary causes limiting the effect of immunotherapy, in addition, the special peripheral immunosuppression of glioma may also compromise the therapeutic efficacy of immunotherapy.

The IL-6 cytokine family has attracted much attention due to its association with the promotion of immunosuppression of the TME in cancers as a proinflammatory factor (42), and current research on IL-6 family-related therapeutic targets has established that agents targeting IL-6 or the IL-6 receptor not only directly inhibit the growth of tumor cells, but also exerts synergistic effects with tumor immunotherapy. Preliminary studies have suggested that the combined targeting of IL-6 and PD-L1 achieved synergistic effects on inhibiting pancreatic ductal and hepatocellular carcinoma growth in mouse models (43, 44). In pancreatic cancer, the activation of the IL-6/JAK/STAT3 signaling pathway could impair the activation of cytotoxic T lymphocytes and led to a decrease in the efficacy of anti-PD-1 immunotherapy, while the selective JAK1/JAK2 kinase inhibitors (Ruxolitinib) decreased the resistance to anti-PD-1 antibodies in mice (45).

In this study, the low CLCF1 expression subgroup was predicted to be more sensitive to anti-PD1 and anti-CTLA4 immunotherapy (with a lower TIDE score). However, in the validation cohorts (GSE78220 and IMvigor210), the low CLCF1 expression group achieved a longer OS and a higher proportion of effective responses to immunotherapy. Moreover, the better immunotherapeutic effect was based on the higher expression of PD-L1, which suggested that CLCF1 as a therapeutic target may also cooperate with anti-PD-L1 immunotherapy;

At present, studies investigating IL-6 family members in glioma have mainly focused on IL-6 and its related pathways, and few studies have involved other IL-6 family members. Due to the shared receptor signaling pathway, IL-6 family members are considered to possess similar activity as IL-6, and IL-6 usually is considered the representative member of the IL-6 family in different studies. However, more recent studies have determined that some members of the IL-6 family exert different or even opposite activities (46, 47), which means that there are still significant functional differences for each family member. In our study, CLCF1 not only showed more accurate and broader prognostic predictive ability than other IL6 family members in the gliomas data cohort (**Supplementary Tables S7**, **S8**) but also had better sensitivity to anti-PD-L1 treatment than other family members (**Supplementary Table S9**). This result indicated that CLCF1 might be more representative than IL-6 in some aspects like predictive value for prognosis and immunotherapy, which also substantiated that CLCF1 possessed important research value in glioma.

There are some limitations to be considered in this study. First, the data analyzed in our study were obtained from public datasets. The predicted results of immunotherapy were verified in retrospective rather than prospective cohorts, and since no appropriate glioma treatment cohort dataset is available, the data of the validation cohorts were extracted from other tumors. Secondly, no appropriate anti-CTLA4 immunotherapy cohort was obtained for validation. Finally, the conclusions obtained from limited bioinformatics analysis are insufficient, and further verification of our findings is needed in more comprehensive experimental and clinical studies.

In conclusion, this study demonstrates that CLCF1 has extensive prognostic significance in gliomas, and its overexpression correlates with immunosuppression and poor prognosis. As a promising target related to immunotherapy outcomes, CLCF1 has the potential of directly inhibiting tumor growth and synergism with immunotherapy.

## Data Availability Statement

The datasets presented in this study can be found in online repositories. The names of the repository/repositories and accession number(s) can be found in the article/**Supplementary Material**.

## Ethics Statement

The studies involving human participants were reviewed and approved by Medical Ethics Committee of the Second Affiliated Hospital of Nanchang University (Nanchang, China). The patients/participants provided their written informed consent to participate in this study.

## Author Contributions

XGZ, KH, and JL were the sponsors of the study. QJ, ZT, PW, and XAZ assisted in the collection of brain tissue samples and the sorting of clinical data. YJ was responsible for collecting and analyzing public data, completing experiments, drawing charts, and writing manuscripts. XGZ and KH reviewed and revised the article. All authors contributed to the article and approved the submitted version.

## Funding

This study was supported by the National Natural Science Foundation of China (grant nos. 81960456, 82002660, 81760445,82172989, and 81760446) and Introduced and Jointly Built High-end R&D Institute of Jiangxi (20203CCH45008).

## Conflict of Interest

The authors declare that the research was conducted in the absence of any commercial or financial relationships that could be construed as a potential conflict of interest.

## Publisher’s Note

All claims expressed in this article are solely those of the authors and do not necessarily represent those of their affiliated organizations, or those of the publisher, the editors and the reviewers. Any product that may be evaluated in this article, or claim that may be made by its manufacturer, is not guaranteed or endorsed by the publisher.
