# Supplementary figures and images for: CLCF1 Is a Novel Potential Immune-Related Target With Predictive Value for Prognosis and Immunotherapy Response in Glioma
Source: Front Immunol. 2022 Feb 21;13:810832. doi: 10.3389/fimmu.2022.810832 (PMC8898905; doi:10.3389/fimmu.2022.810832)

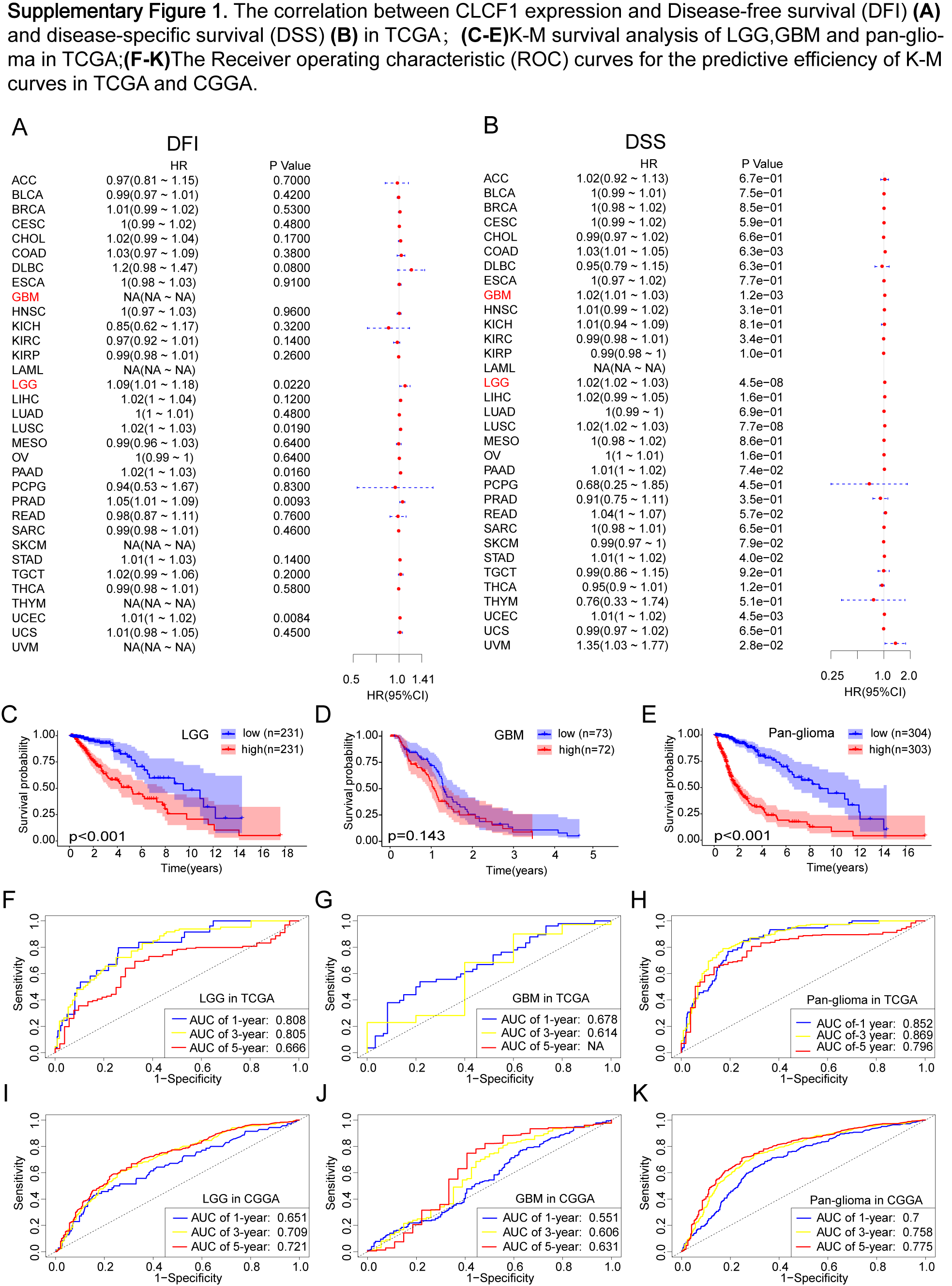

Supplement: Supplementary file 1 [file Image_1.tif]

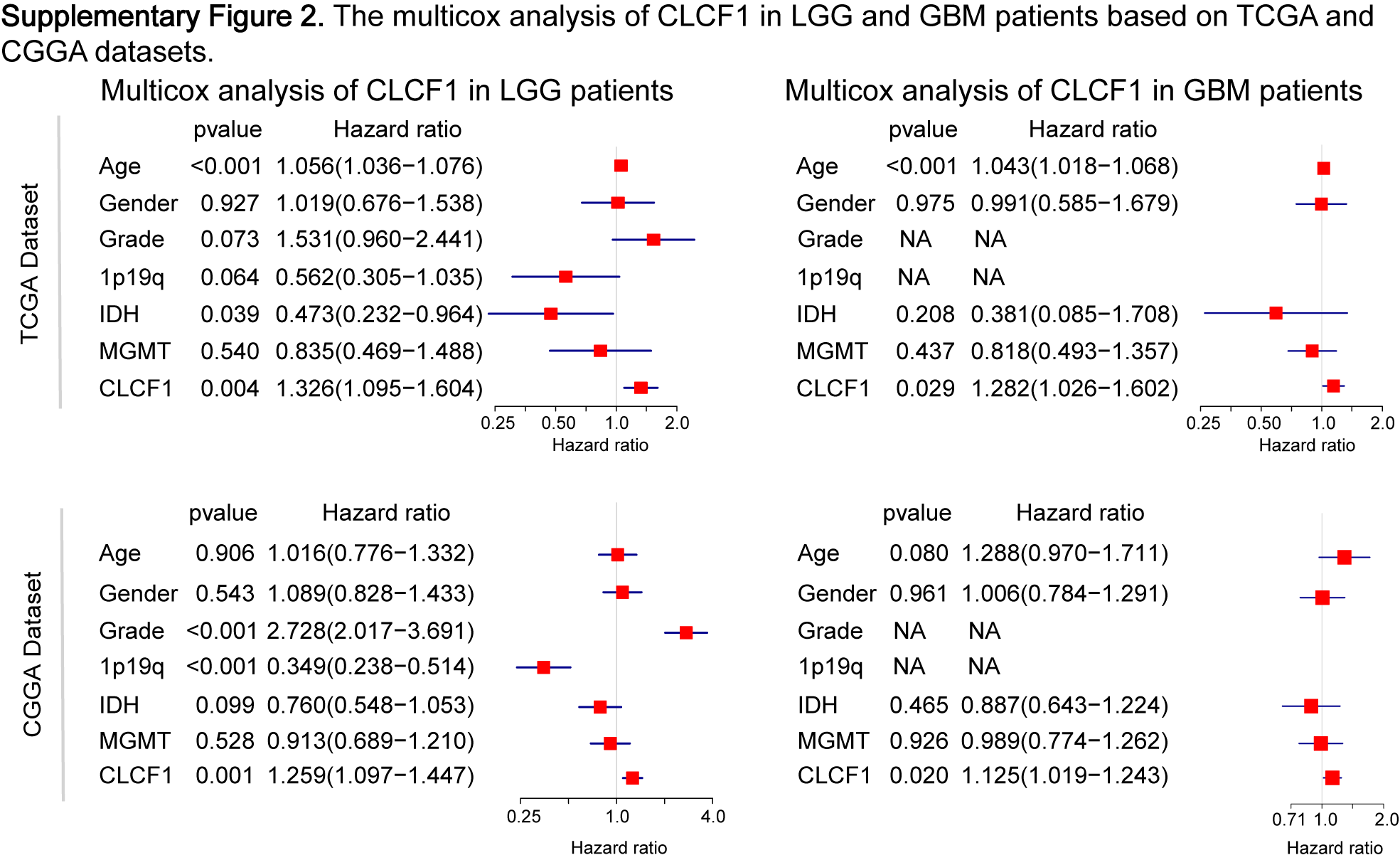

Supplement: Supplementary file 2 [file Image_2.tif]

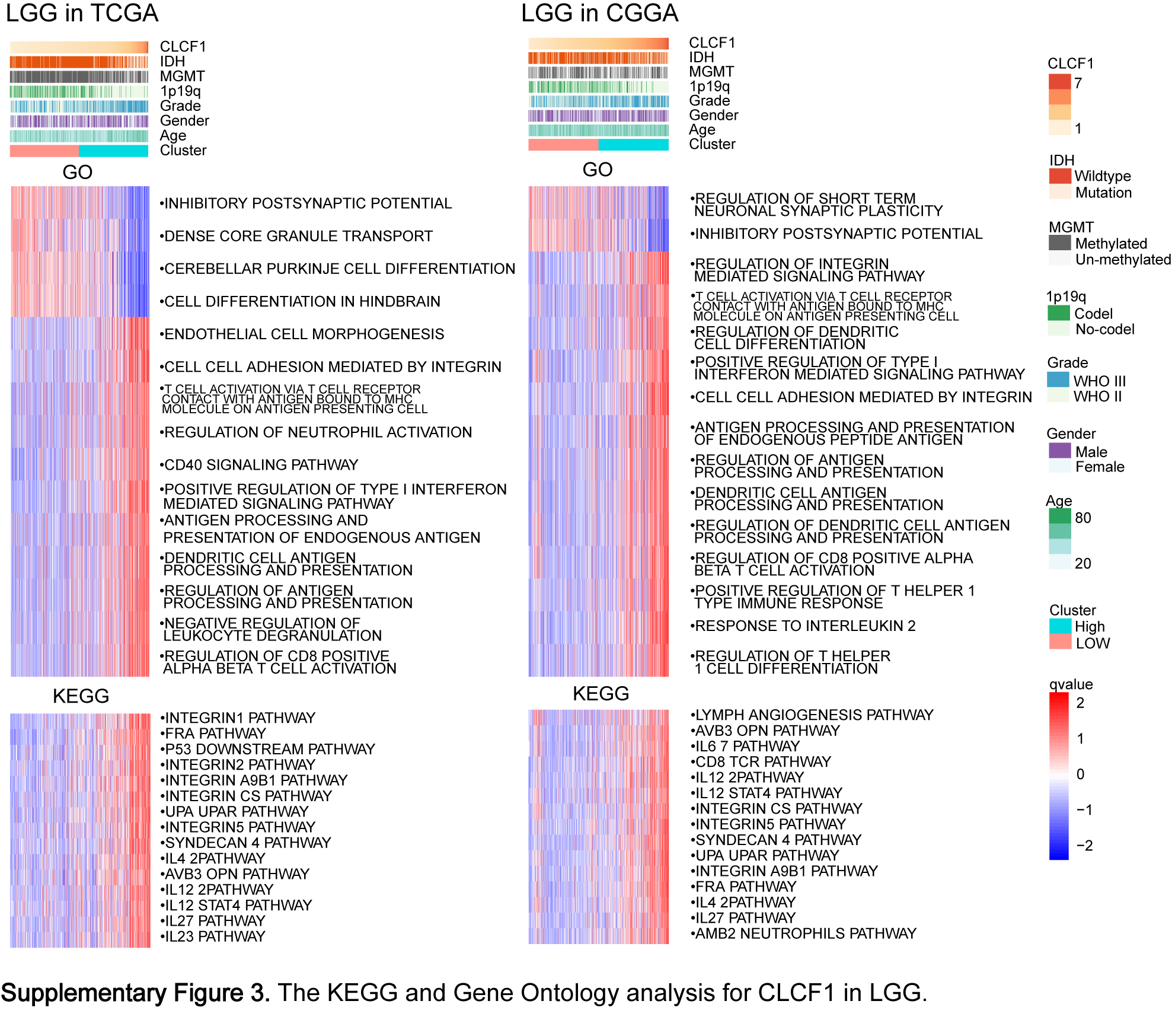

Supplement: Supplementary file 3 [file Image_3.tif]

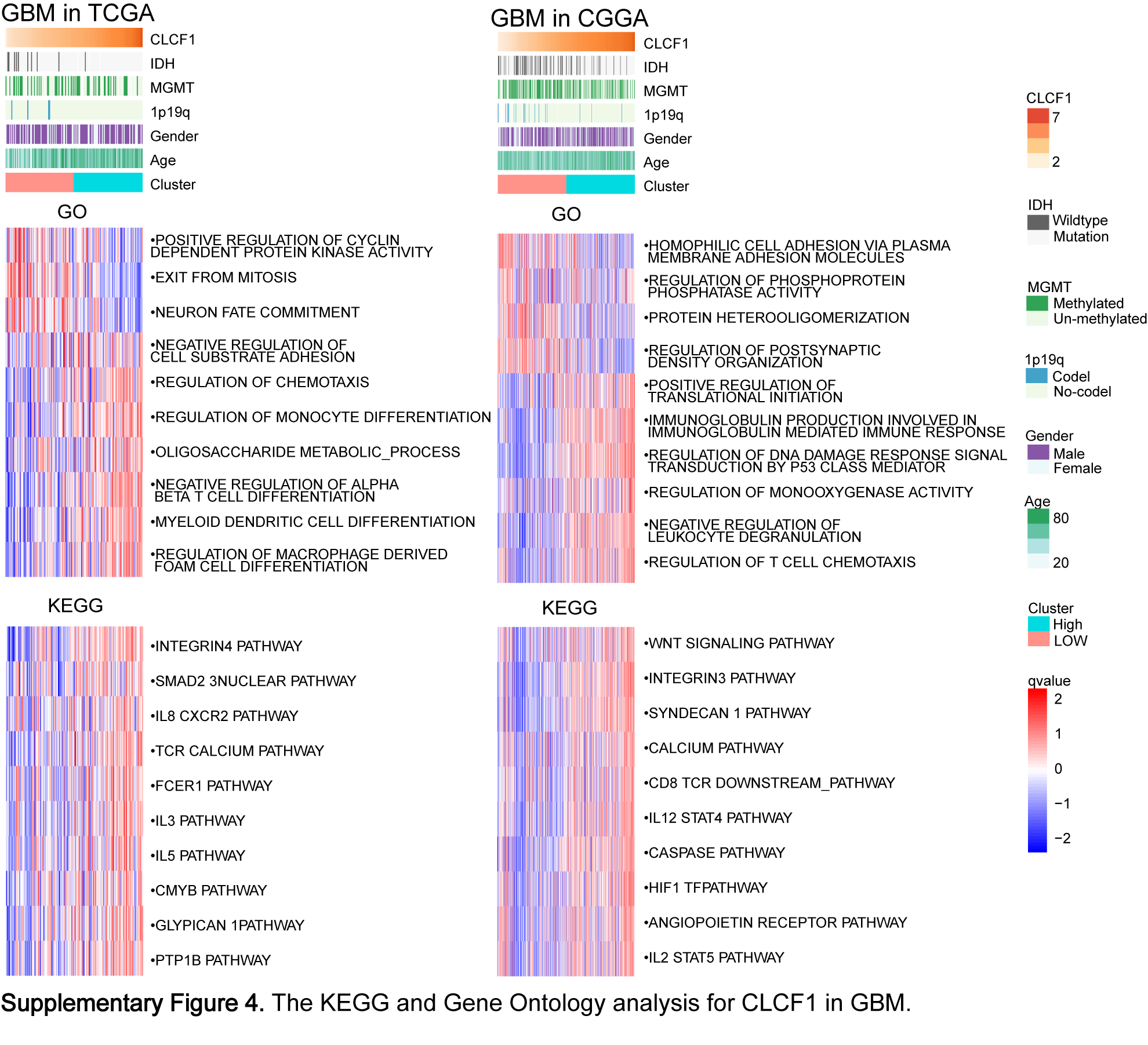

Supplement: Supplementary file 4 [file Image_4.tif]

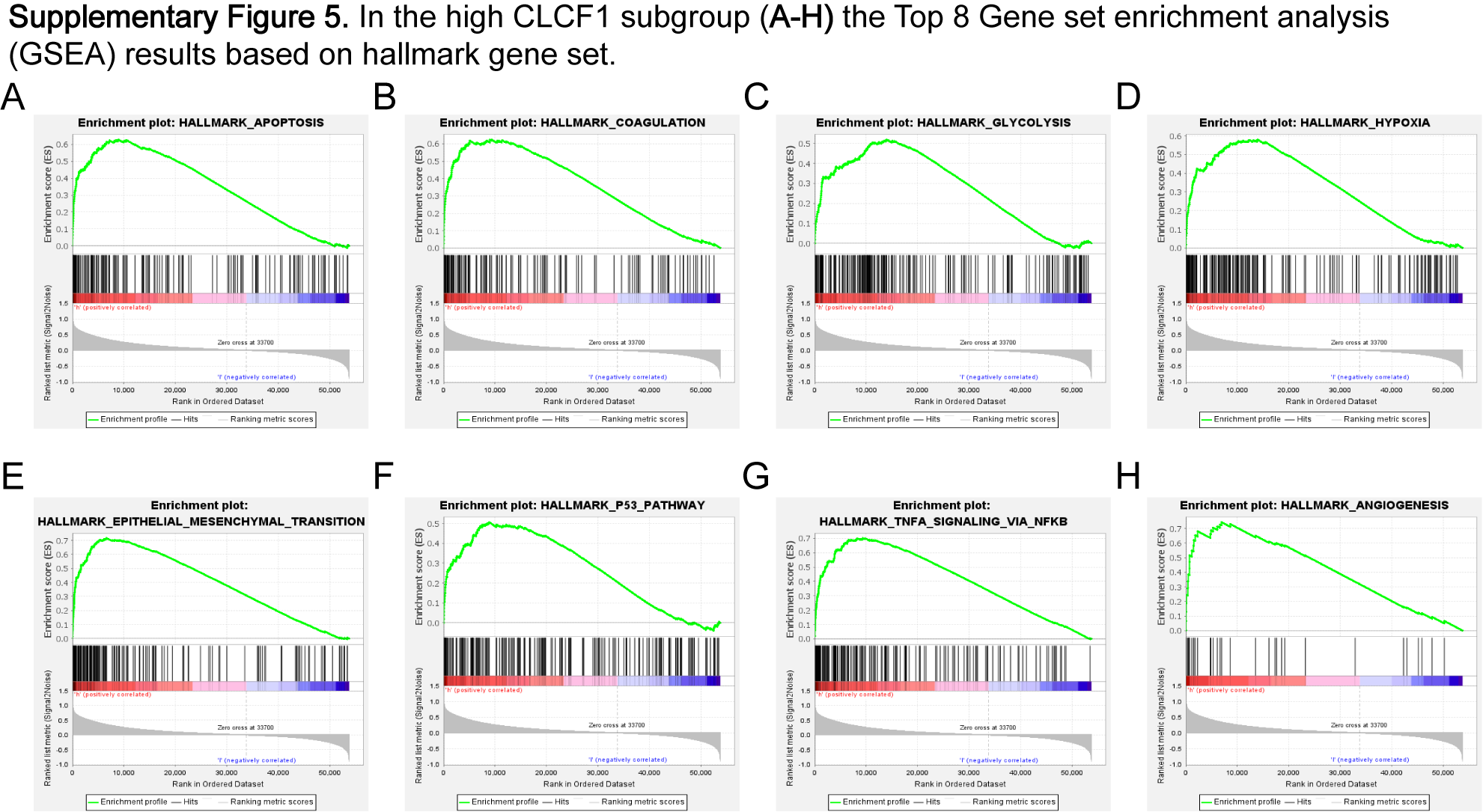

Supplement: Supplementary file 5 [file Image_5.tif]

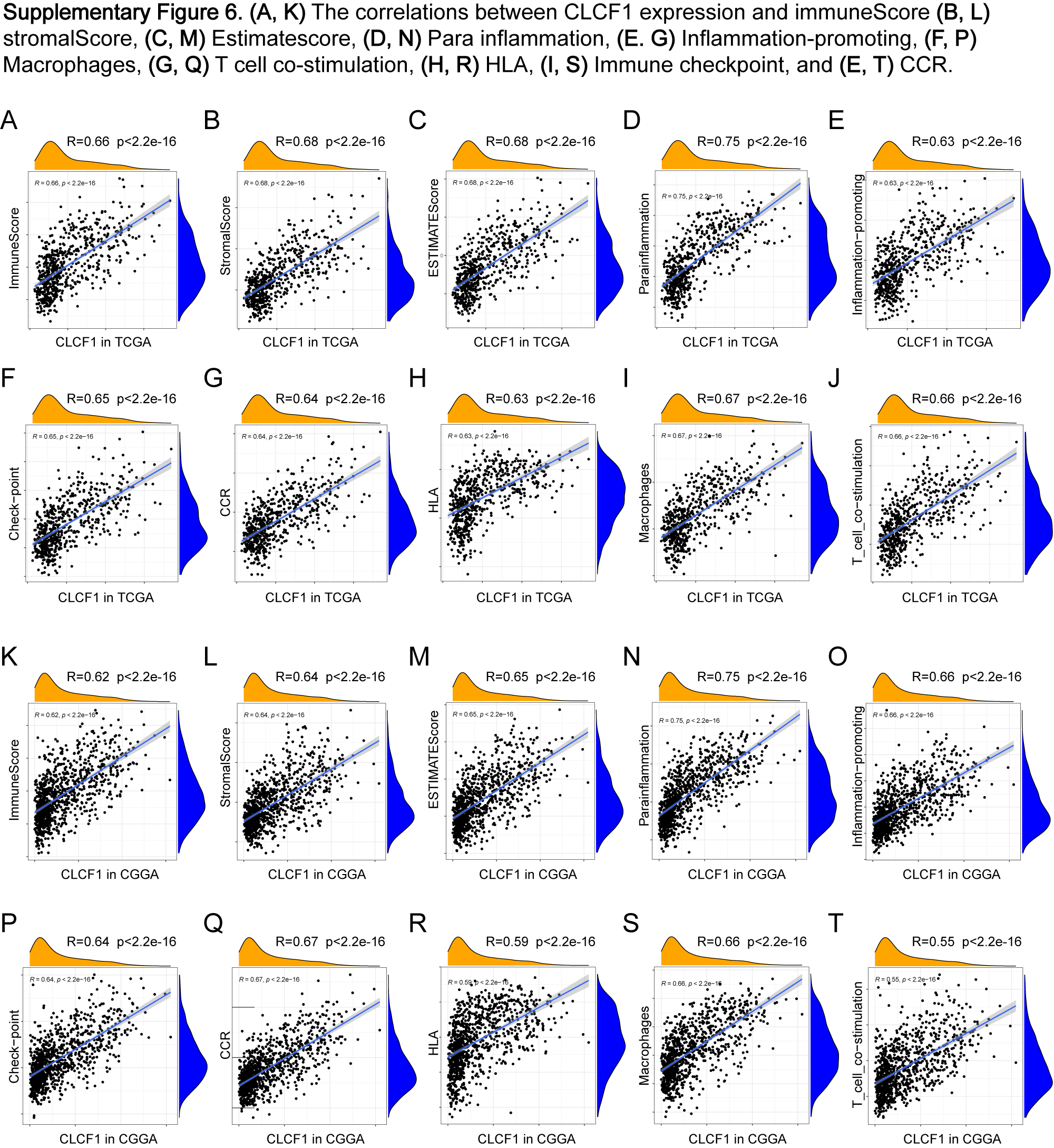

Supplement: Supplementary file 6 [file Image_6.tif]

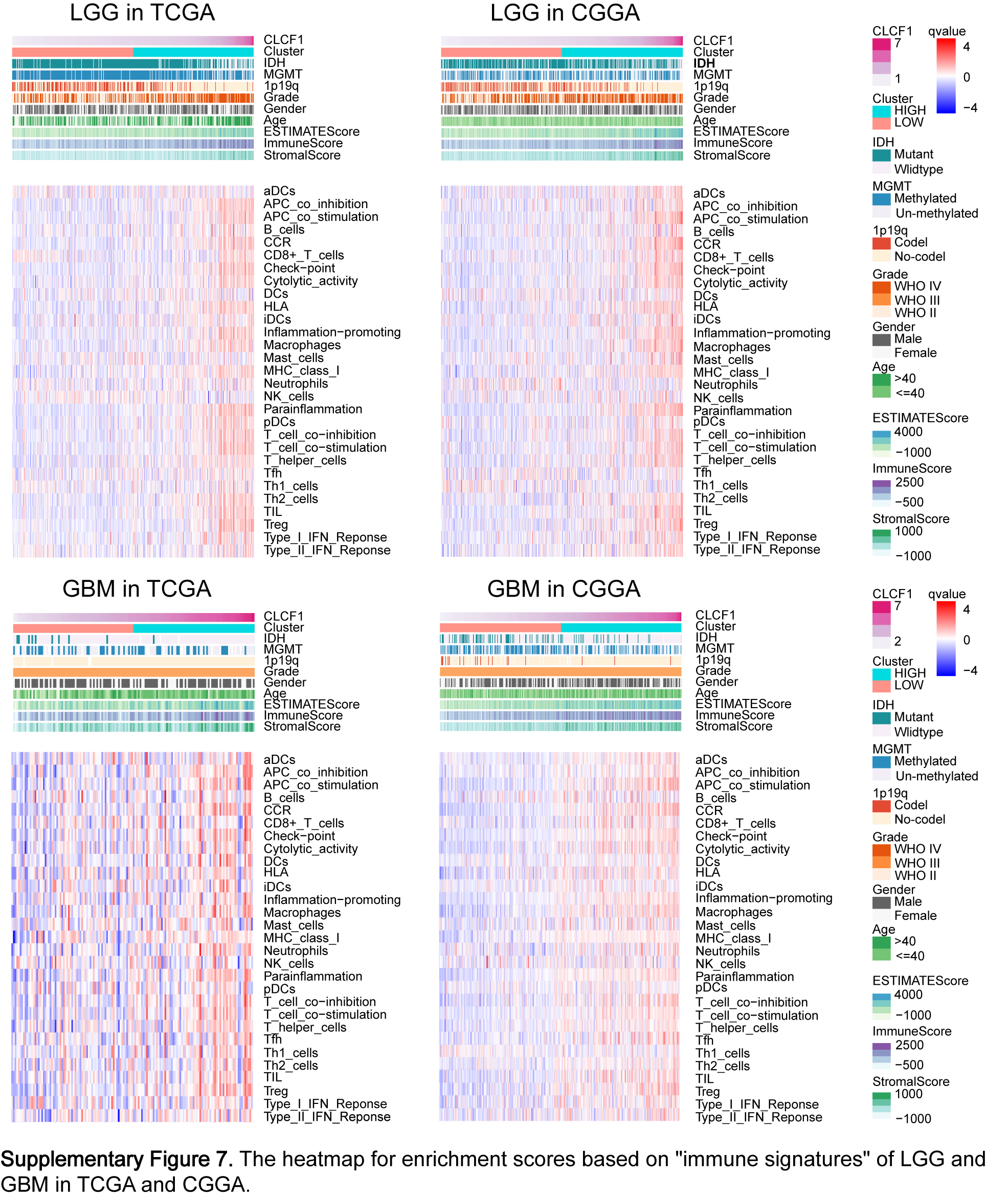

Supplement: Supplementary file 7 [file Image_7.tif]

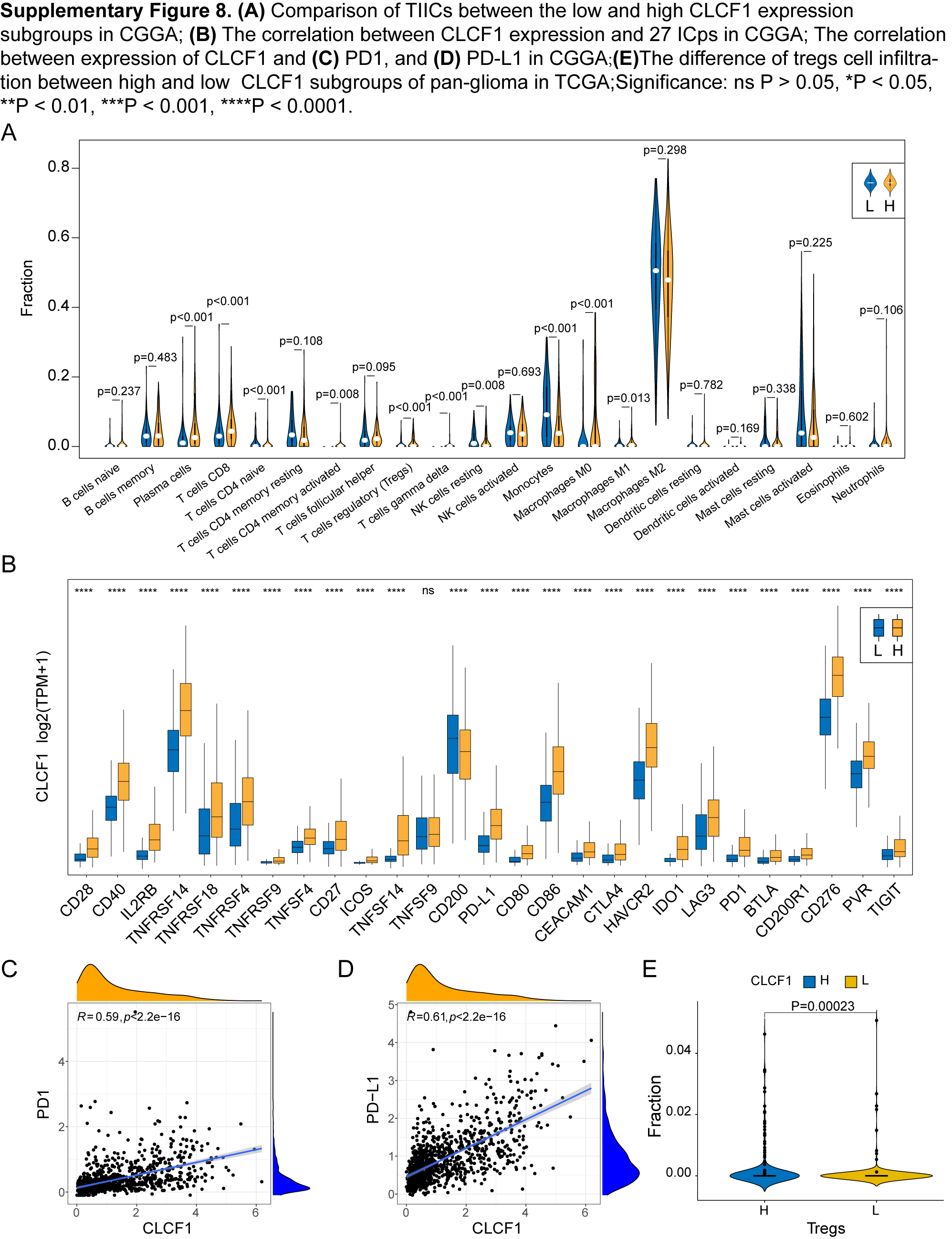

Supplement: Supplementary file 8 [file Image_8.tif]

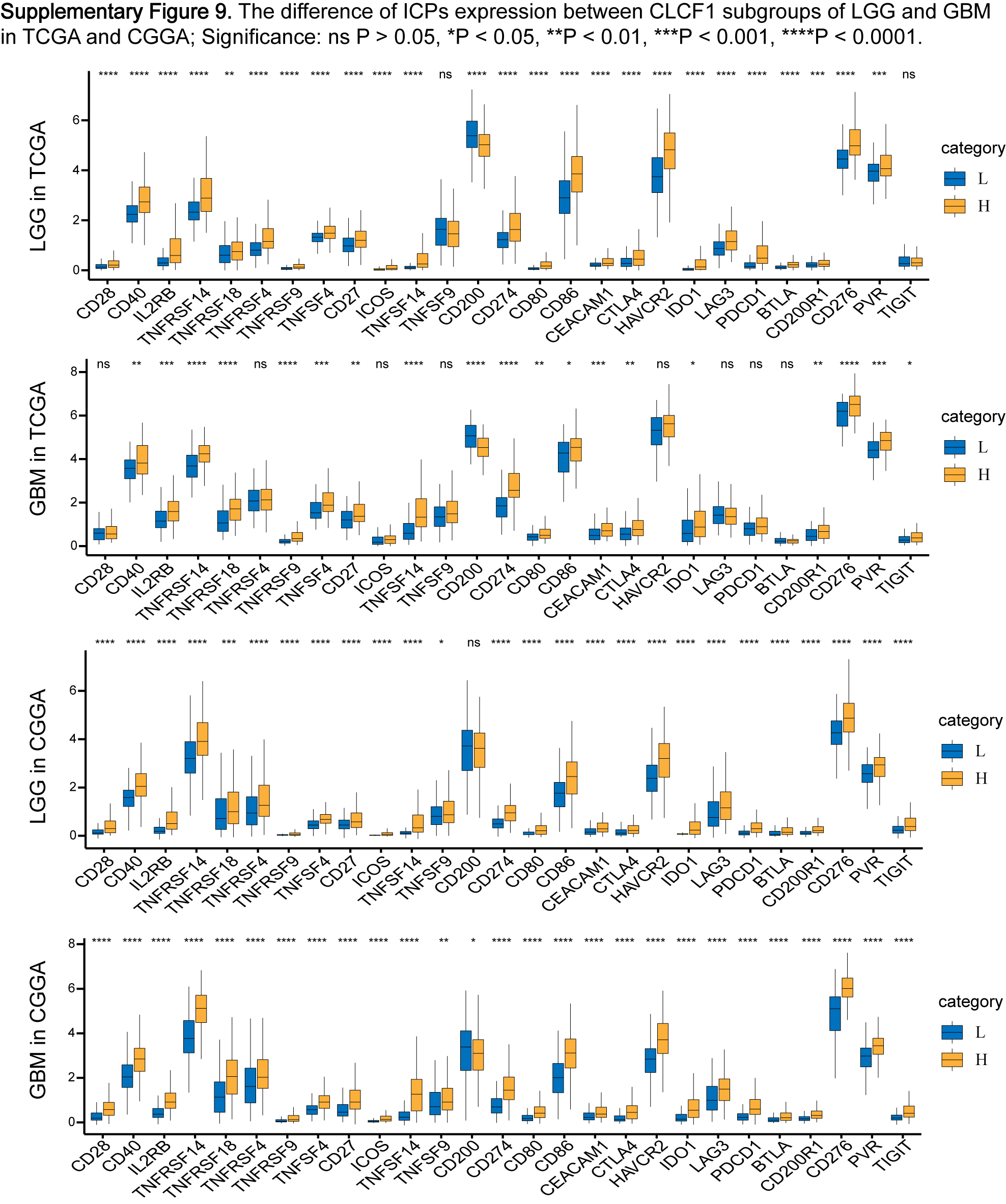

Supplement: Supplementary file 9 [file Image_9.tif]

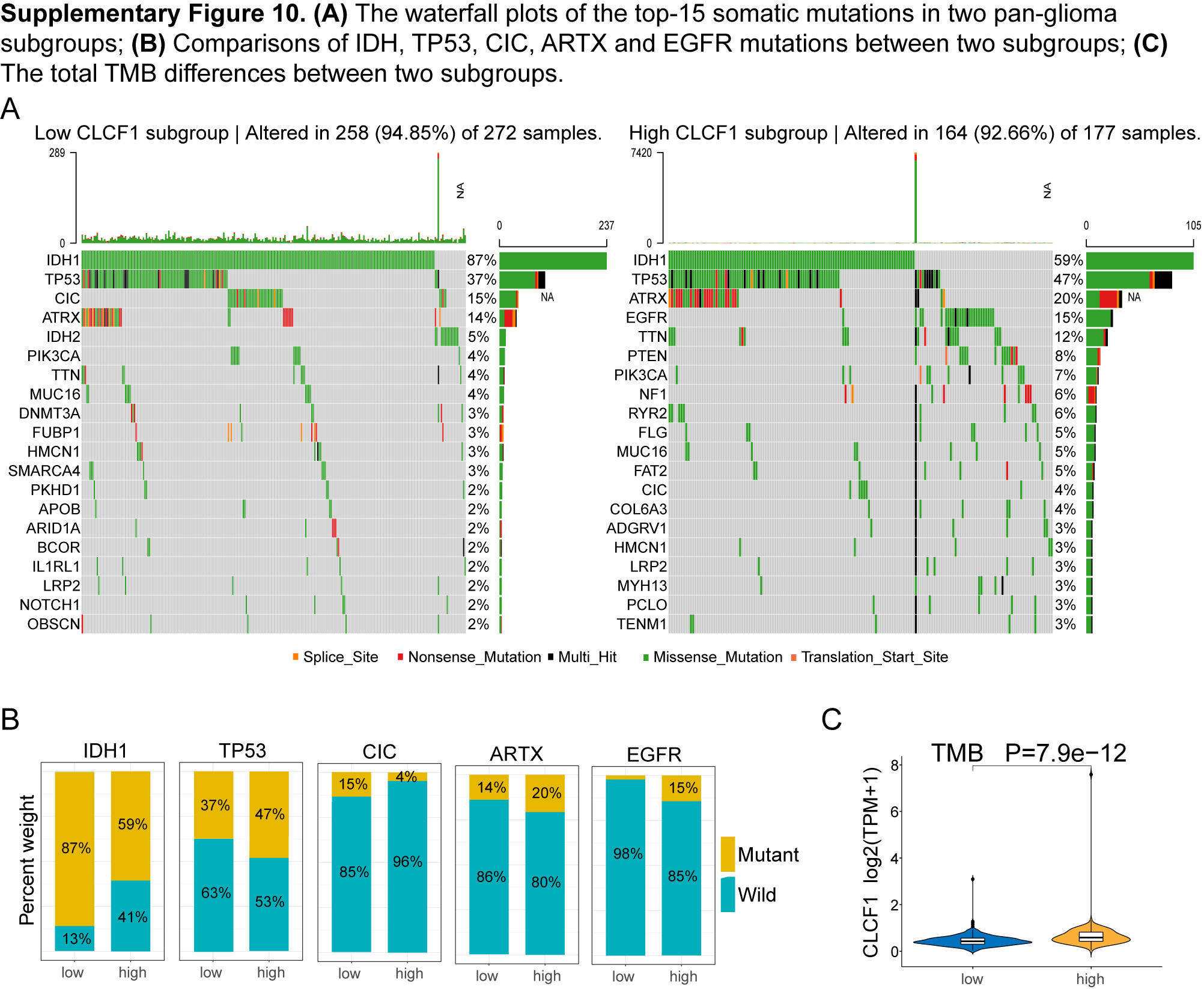

Supplement: Supplementary file 10 [file Image_10.tif]

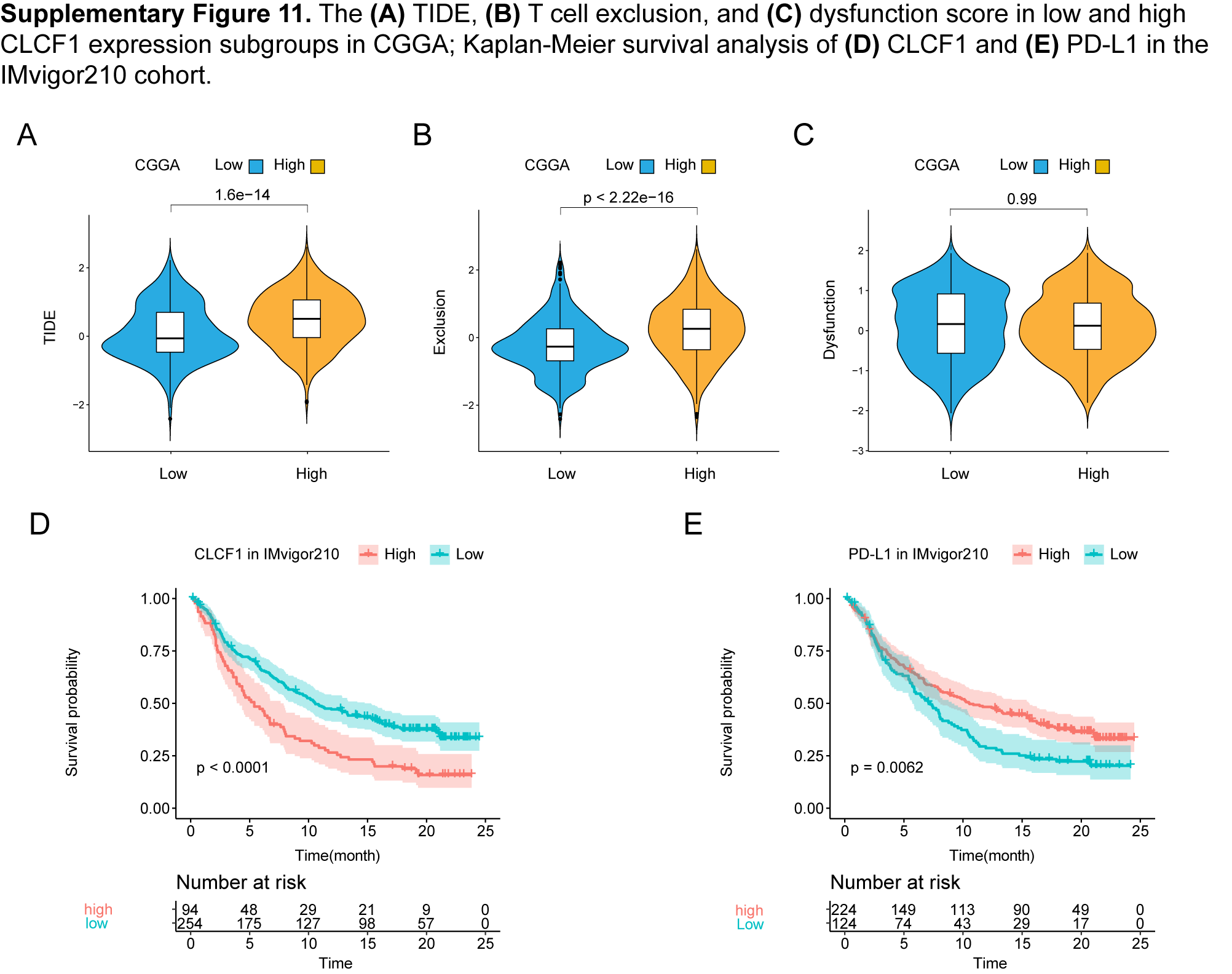

Supplement: Supplementary file 11 [file Image_11.tif]
